# Changes in attitudes, beliefs, and experiences related to pregnancy during Graduate Medical Education training from 2005 to 2021

**DOI:** 10.17305/bb.2023.9865

**Published:** 2024-06-01

**Authors:** Lindsay Warner, Lindsay Hunter Guevara, Andrea Watson, Sara Farmer, Ramila Mehta, Jason Homme

**Affiliations:** 1 Department of Anesthesiology and Perioperative Medicine, Mayo Clinic, Rochester, USA; 2 Department of Oncology, Essentia Health, Duluth, USA; 3 Department of Clinical Trials and Biostatistics, Mayo Clinic, Rochester, USA; 4 Department of Community Pediatric and Adolescent Medicine, Mayo Clinic, Rochester, USA

**Keywords:** Maternity leave, parental leave, pregnancy, residency, training

## Abstract

Today, 50% of medical students are women, and residency and fellowship training years overlap with peak times for starting families. The authors describe attitudes toward pregnancy during residency and fellowship and report pregnancy rates and complications for female residents and resident partners across several decades. A web-based survey was emailed to 1057 residents in 2005 (period 1) and 1860 residents in 2021 (period 2). Anonymous surveys were sent to all trainees, including pregnant trainees, affected co-trainees, and trainee partners. Resident attitudes and pregnancy characteristics were compared between groups using the chi-square (χ2) test for categorical variables and the Kruskal–Wallis test for ordinal variables. A total of 442 residents (41.8%) responded to the 2005 survey, and 525 (28.2%) responded to the 2021 survey. Most residents who covered for a pregnant resident had positive feelings about covering for their colleagues during both time periods, although more positive attitudes were present during period 2. Only about 10% of residents received compensation for their coverage during both time periods. Among residents with a pregnancy during training (i.e., themselves or partners), most characterized having a baby in training as “somewhat difficult” or “very difficult” at both time periods. Pregnancy complication rates were 33% and 44% for training years 2005 and 2021. As medical education evolves, training programs should be proactive in creating structured support systems for pregnant residents and resident partners to minimize adverse maternal and fetal outcomes and to improve training programs. Future studies are needed to elucidate the causality of higher-than-expected pregnancy complication rates.

## Introduction

Fifty years ago, less than 10% of medical students were women. Today, nearly 50% of all entering medical students are women [1], and more than 50% of residency slots are filled by women in the specialties of pathology (51.0%), family practice (51.9%), psychiatry (52.7%), dermatology (60.3%), pediatrics (69.1%), and obstetrics and gynecology (74.5%) [2]. Because continued medical education typically occurs during childbearing years, many residents and fellows begin families during training. Having a baby is ranked among the top ten of life’s most stressful events [3]. The effect of pregnancy on resident scheduling can be onerous, affecting not only the resident but also colleagues, staff, and patients. By law, training programs are required to accommodate pregnant and postpartum residents by balancing safety, call coverage, and program morale. However, program accommodations can vary greatly across institutions, depending on each program’s specific institutional residency review committee and specialty board requirements for the duration of training and allowable time away from training [4]. Little is known about current pregnancy rates and the accompanying issues among medical residents. In the early 2000s, approximately 33% of all female residents were pregnant during training, with higher pregnancy rates for married female residents [5–8]. This percentage, however, may have changed as more women have entered medicine, and attitudes among residents regarding pregnancy may also have changed. In addition, certain complications have been reported more commonly for pregnant residents than for pregnant partners of male residents, including preterm labor [5, 9], intrauterine growth retardation [10], small for gestational age [5, 7], and pregnancy-induced hypertension [5, 7, 11]. Indeed, many work characteristics of residency training, including physically demanding work, prolonged standing, shift and night work, and high levels of fatigue correlate with increased complications during pregnancy [12].

The objectives of our study were to characterize and compare pregnancy-related attitudes of residents and report the rates of pregnancy and complications of pregnancy during residencies at a large Midwestern teaching institution, at two time points spanning several decades.

## Materials and methods

An anonymous web-based survey instrument was developed and distributed via email at two distinct time periods as detailed in supplemental survey items. In 2005 (period 1), 1057 residents and fellows (hereafter referred to collectively as residents) at a Midwestern training institution were sent the survey, and in 2021 (period 2), 1860 residents at the same institution were sent the survey. The institution’s Survey Research Center assisted in the design and distribution of the survey. Qualtrics programming (Qualtrics XM) was used to collect response data. Three reminders were sent during the survey window. Participation was voluntary but incentivized with a random drawing for local business gift cards, held after the survey was completed. To maintain anonymity, email contacts for eligible participants, which included all residents enrolled in the Mayo Clinic School of Graduate Medical Education (MCSGME), were provided by the MCSGME office directly to survey research staff. A copy of the survey instrument can be obtained by contacting the corresponding author (L.L.W.).

All respondents were asked about covering for a pregnant resident; awareness of established maternity, paternity, and adoptive leave policies; and the overall effect of pregnancy on their programs. Suggestions for changes that would improve the experience of pregnancy/adoption were also solicited. Respondents who experienced pregnancy (themselves or their partner) were also queried about complications, time off from work, and stressors related to the pregnancy. The survey wording was nearly identical for the two periods. A few minor changes were made to provide inclusive language in the demographic section and some updated terms for obstetric diagnoses. The pregnancy complication responses “preeclampsia/eclampsia” and “pregnancy-induced hypertension” from the period 1 survey were condensed under the umbrella term “hypertensive disorders of pregnancy,” and the pregnancy complication response “placenta previa” from the period 1 survey was updated and included under the umbrella term “placental abnormalities” in the period 2 survey.

A comparison was made between surgical and non-surgical trainees. Surgical trainees tend to spend more time on their feet and have less immediate access to food and water throughout their day.

### Ethical statement

The Institutional Review Board exempted our study on 10/8/2021 (IRB 21-005474). The Mayo Clinic Institutional Review Board and the MCSGME approved the survey before distribution.

### Statistical analysis

Resident demographic information, attitudes about pregnancy, and pregnancy characteristics were summarized as numbers and percentages. These data sets were then compared across groups with the chi-square (χ2) test for categorical variables and the Kruskal–Wallis test for ordinal variables. Pregnancies and complications were summarized on a per-resident basis unless otherwise specified. Because the sample size was small for the group reporting “undisclosed/other gender” and this response option was only used in the period 2 survey, summary statistics were reported, but comparisons across other genders were not made for this group. Statistical analyses were performed using the SAS package (version 9.4, SAS Institute Inc.).

## Results

### Resident cohort characteristics

**Figure 1.**
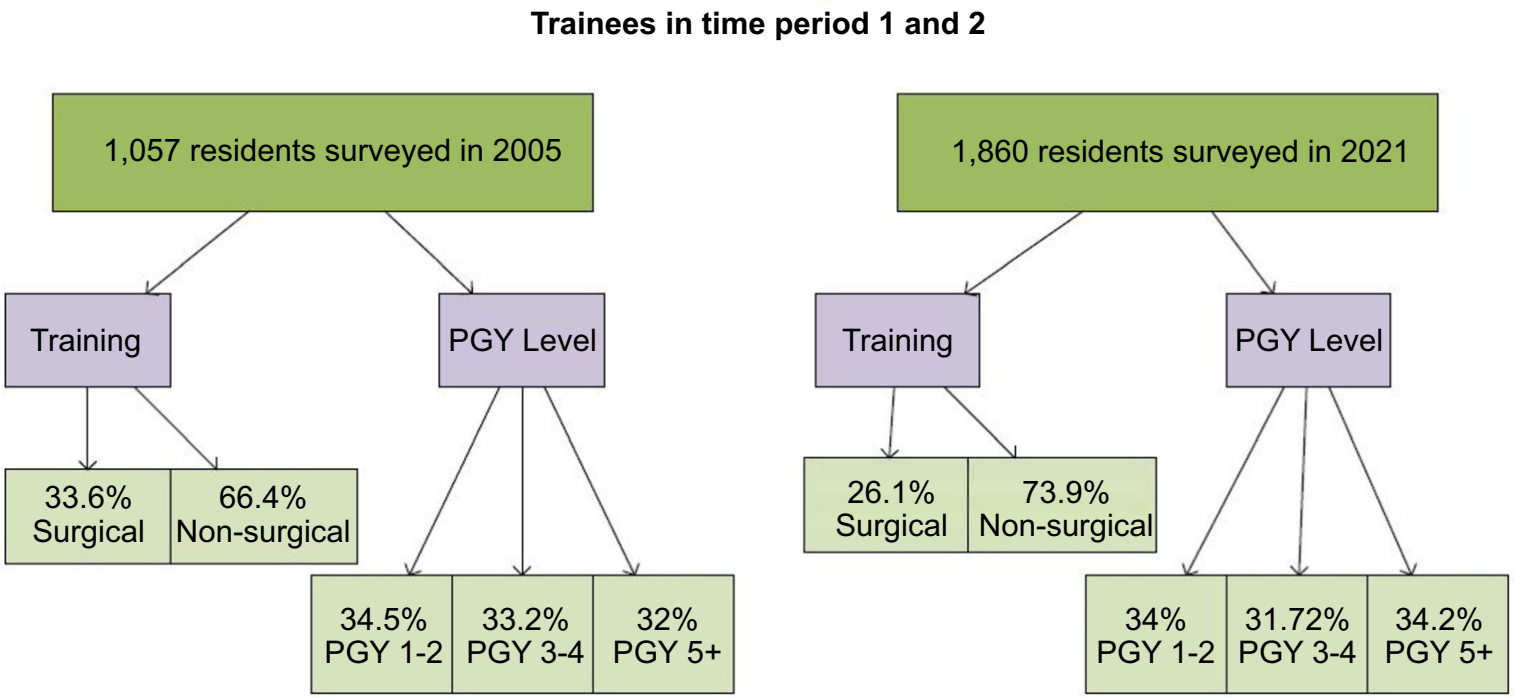
**Trainees in time period 1 and 2 summarized.** PGY: Postgraduate year.

There were 1057 residents surveyed in 2005 (41.8% response rate), and 1860 residents in 2021 (28.2% response rate). Detailed information regarding the type of training and training level are shown in Figure 1 and additional characteristics of resident cohorts are shown in Table 1.

**Table 1:** Characteristics of respondents in the two time periods (*N* ═ 967)^a^

	**No. (%)**	
**Demographic information**	**Period 1 respondents (*n* ═ 442)**	**Period 2 respondents (*n* ═ 525)**
*Gender*		
Male	246 (55.7)	220 (41.9)
Female	195 (44.1)	273 (52.0)
Undisclosed/other	N/A	32 (6.1)
Not specified	1 (0.2)	0 (0)
*Type of training*		
Surgical	136 (30.8)	114 (21.7)
Nonsurgical	280 (63.3)	388 (73.9)
Both	18 (4.1)	0 (0)
Not specified	8 (1.8)	23 (4.4)
*Level of training*		
PGY 1-2	155 (35.1)	178 (33.9)
PGY 3-4	155 (35.1)	184 (35.0)
PGY 5+	130 (29.4)	141 (26.9)
Not specified	2 (0.4)	22 (4.2)

^a^Characteristics of the survey respondents were obtained anonymously via self-report. N/A: Not applicable, not included as an item in the survey administered in period 1; PGY: Postgraduate year.

### Attitudes among residents

Attitudes about the impact pregnant or adopting residents had on the program were positive and residents also reported being influenced by a program’s support (or lack of) for pregnancy/adoption when choosing their training program (Table 2). However, about 25% of residents were unaware of any parental leave policies in their program. Trainees most wanted compensation for covering for fellow trainees (57.7% and 60%) (Table 2).

**Table 2:** Attitudes about pregnancy in the two time periods (*N* ═ 967)^a^

**Gender by year**	**2005 Male (*N* ═ 246)**	**2021 Male (*N* ═ 220)**	**2005 Female (*N* ═ 195)**	**2021 Female (*N* ═ 273)**	**2021 Other (*N* ═ 32)**
*Aware of the written policies (%)*					
Maternity leave	175 (71.1)	141 (64.1)	149 (76.4)	177 (64.8)	11 (34.4)
Paternity leave	160 (65.0)	138 (62.7)	105 (53.8)	131 (48.0)	11 (34.4)
Adoption leave	63 (25.6)	41 (18.6)	77 (39.5)	65 (23.8)	6 (18.8)
Not aware of any of the above	59 (24.0)	59 (26.8)	44 (22.6)	95 (34.8)	9 (28.1)
*Impact that pregnant/adopting residents have on program (%)*					
Very positive impact	46 (18.7)	79 (35.9)	34 (17.4)	79 (28.9)	8 (25.0)
Somewhat positive impact	53 (21.5)	62 (28.2)	54 (27.7)	68 (24.9)	2 (6.3)
No impact	77 (31.3)	62 (28.2)	62 (31.8)	82 (30.0)	4 (12.5)
Somewhat negative impact	63 (25.6)	15 (6.8)	42 (21.5)	41 (15.0)	2 (6.3)
Very negative impact	6 (2.4)	2 (0.9)	2 (1)	3 (1.1)	4 (12.5)
Not specified	1 (0.4)	0 (0)	1 (0.5)	0 (0)	12 (37.5)
*Changes that could improve pregnancy/adoption experience during training for trainees or the program (%)*					
Clear written maternity or paternity or adoption	113 (45.9)	101 (45.9)	94 (48.2)	151 (55.3)	3 (9.4)
Established call coverage for pregnant trainees	124 (50.4)	92 (41.8)	114 (58.5)	154 (56.4)	6 (18.8)
Compensation for covering trainees	139 (56.5)	132 (60.0)	116 (59.5)	176 (64.5)	7 (21.9)
Increase the number of trainees per year	40 (16.3)	62 (28.2)	43 (22.1)	87 (31.9)	4 (12.5)
Reduce the call schedule for pregnant trainees	66 (26.8)	84 (38.2)	82 (42.1)	140 (51.3)	7 (21.9)
Develop special electives for new mothers/fathers	103 (41.9)	97 (44.1)	102 (52.3)	163 (59.7)	6 (18.8)
Provide temporary coverage from practitioners other than trainees	N/A	92 (41.8)	N/A	138 (50.5)	8 (25.0)
Other	31 (12.6)	19 (8.6)	20 (10.3)	25 (9.2)	3 (9.4)
*Support of a program (or lack of) for pregnancy or adoption influenced the choice of training program (%)*					
Greatly influenced	8 (3.3)	35 (15.9)	27 (13.8)	58 (21.2)	3 (9.4)
Somewhat influenced	34 (13.8)	49 (22.3)	48 (24.6)	76 (27.8)	1 (3.1)
Very little influence	23 (9.3)	36 (16.4)	25 (12.8)	37 (13.6)	2 (6.3)
No influence	179 (72.8)	98 (44.5)	94 (48.2)	102 (37.4)	6 (18.8)
Not specified	2 (0.8)	2 (0.9)	1 (0.5)	0 (0.0)	20 (62.5)

^a^Used with permission of Mayo Foundation for Medical Education and Research. N/A: Not applicable, not included as an item in the survey administered in period 1.

### Attitudes among residents who covered for pregnant residents

Among all resident respondents, half reported having covered for a pregnant resident during their training, (224 [50.7%] in period 1 and 263 [50.1%] in period 2). Most residents expressed positive feelings about covering shifts. While only roughly 10% of residents reported being compensated (Table 2).

### Attitudes among residents who had a pregnancy during training

For residents who had a pregnancy or a partner with a pregnancy during training, most characterized the experience as “somewhat difficult” or “very difficult” and most often cited “physical demands of work” (Table 2). Fortunately, more residents ranked their program as supportive of pregnancy during period 2 than period 1. Stratified by specialty, female surgical residents were more likely to report “having a baby during training” to be “very difficult” due to the “physical demands of work”. When stratified by training year, more female residents in period 1 reported having a baby during training as more difficult, increasing by training year, although results were similar across the training year for period 2.

### Complications of pregnancy

Of the 442 survey respondents in period 1197 (44.6%) reported a pregnancy, for themselves or their partner during residency, with a total of 270 pregnancies reported. Fewer pregnancies, 222 total, were reported in period 2. During both periods, the percentage of men who fathered a child during training was larger than the percentage of female residents who were pregnant during training (period 1: 134/246 [54.5%] vs 63/195 [32.3%]; period 2: 85/220 [38.6%] vs 61/273 [22.3%]). When considering complications among pregnant residents or partners of male residents, pregnancy complication rates were 36% and 38% for training years 2005 and 2021, respectively. Complication rates for pregnancy were similar between female residents and partners of male residents in period 1 (22 [34.9%] vs 48 [35.8%]) though rates were higher for female residents than for partners of male. Preterm labor, miscarriage/stillborn, and hypertensive disorders of pregnancy were the most common complications reported in both gender groups across both periods (Table 3).

**Table 3:** Complications of pregnancy among pregnant residents or partners of male residents (*N* ═ 967)^a^

	**No. (%)**				
**Complication**	**Period 1 (*n* ═ 197)**		**Period 2 (*n* ═ 156)**		
	**Women (*n* ═ 63)**	**Partner^b^ (*n* ═ 134)**	**Women (*n* ═ 61)**	**Partner (*n* ═ 85)**	**Undisclosed/other gender (*n* ═ 10)**
*Pregnancies during training*					
1	49 (77.8)	88 (65.7)	46 (75.4)	52 (61.2)	6 (60.0)
2 or more	14 (22.2)	46 (34.3)	15 (24.6)	33 (38.8)	4 (40.0)
At least 1 pregnancy with a complication	22 (34.9)	48 (35.8)	27 (44.3)	29 (34.1)	3 (30.0)
*Complications*					
Preterm labor	7 (11.1)	13 (9.7)	6 (9.8)	3 (3.5)	1 (10.0)
Hyperemesis	N/A	N/A	1 (1.6)	2 (2.4)	0 (0)
Hypertensive disorders of pregnancy	4 (6.3)	4 (3.0)	6 (9.8)	6 (7.1)	0 (0)
Miscarriage or stillbirth	6 (9.5)	11 (8.2)	10 (16.4)	6 (7.1)	1 (10.0)
Gestational diabetes	0 (0)	1 (0.7)	3 (4.9)	5 (5.9)	0 (0)
Placental abnormalities	0 (0)	2 (1.5)	5 (8.2)	1 (1.2)	0 (0)
Multiple fetuses	0 (0)	7 (5.2)	2 (3.3)	2 (2.4)	0 (0)
Other	9 (14.3)	22 (16.4)	7 (11.5)	12 (14.1)	2 (20.0)

^a^One resident did not respond to the gender question during period 1 and was omitted from the stratified analysis. ^b^Partner of a male resident. N/A: Not applicable, not included as an item in the survey administered in period 1.

Only a small number of respondents reported more than one pregnancy during their training (Table 3). Complication rates were similar between women in surgical and nonsurgical specialties in period 1 (4 [33.3%] vs 16 [33.3%]). However, complication rates were higher in surgical specialties than in nonsurgical specialties in period 2 (Table 4).

**Table 4:** Pregnancy complications among residents during training

	**No. (%)**			
**Complication**	**Period 1 (*n* ═ 60)^a^**		**Period 2 (*n* ═ 61)**	
	**Surgical (*n* ═ 12)**	**Nonsurgical (*n* ═ 48)**	**Surgical (*n* ═ 11)**	**Nonsurgical (*n* ═ 50)**
*Pregnancies during training*				
1	10 (83.3)	39 (81.3)	8 (72.7)	38 (76.0)
2 or more	2 (16.7)	9 (18.8)	3 (27.3)	12 (24.0)
At least 1 pregnancy with a complication	4 (33.3)	16 (33.3)	7 (63.6)	20 (40.0)
*Complications*				
Preterm labor	2 (16.7)	4 (8.3)	2 (18.2)	4 (8.0)
Hyperemesis	N/A	N/A	1 (9.1)	0 (0)
Hypertensive disorders of pregnancy	0 (0)	4 (8.3)	0 (0)	6 (12.0)
Miscarriage or stillbirth	0 (0)	4 (8.3)	3 (27.3)	7 (14.0)
Gestational diabetes	0 (0)	4 (8.3)	0 (0)	3 (6.0)
Placental abnormalities	0 (0)	0 (0)	1 (9.1)	4 (8.0)
Other	2 (16.7)	6 (12.5)	2 (18.2)	5 (10.0)

^a^Results for period 1 are based only on those residents who reported their specialty (either surgical or nonsurgical). Residents who reported both a surgical and a nonsurgical specialty were categorized as surgical. Of the 63 female residents who reported a pregnancy, 3 did not report a training specialty. N/A: Not applicable, not included as an item in the survey administered in period 1.

## Discussion

Our main findings showed that pregnancy during training is an important issue for residents and that pregnancy complication rates are roughly a third of surveyed trainees. Similar distributions of men and women among respondents for both time periods indicate that pregnancy during residency training is an important issue for all residents, regardless of gender.

Pregnancy complication rates were substantially higher (33% and 44%) than those in the general population. These rates are higher than the 17% reported rate for the general population [13] although this finding is difficult to interpret owing to the self-reported study design and the lack of a common definition of “complication”. There is a possibility of selection bias since these respondents are medical professionals who may be more apt to seek medical care at the tertiary care center, they practice in. In addition, because they are likely receiving care at a resource-abundant institution, the diagnostics available to them may be more abundant compared to the general population. Preterm labor and miscarriage/stillbirth rates were the most reported pregnancy complications among the residents, which is consistent with a previous report [14]. A future study will be needed to explore the possible cause of this discrepancy of pregnancy complications between resident physicians and the general population.

Overall attitudes regarding coverage for pregnancy-related issues during residency were positive. However, our findings showed an inverse relationship between positive attitudes and years of residency training. Although over half of residents were asked to cover for a coworker, only a small minority received compensation for their coverage. This could be a factor in fostering negative attitudes toward the overall perception of pregnancy during training and level for pregnant residents. Approximately 60% identified compensation for covering residents as a change that would improve the pregnancy experience in residency. Clearly, pregnant residents are concerned about the effects of their pregnancies on their colleagues’ workloads. Although residency programs are currently required to have mechanisms to prevent duty-hour violations, perhaps some of the creativity used by programs to become compliant with duty-hour restrictions could also be used to provide equity for residents asked to cover. A shared backup call system, additional vacation, preferential call scheduling, or simply positive recognition by the program leadership may suffice to improve residents’ morale and ameliorate some of the stress related to scheduling disruptions.

“Physical demands of work” was the most-cited work-related factor during both time periods contributing to stress during pregnancy suggesting that work-related modifications could alleviate some degree of the perceived stress. To allow for decreased call coverage toward the end of pregnancy or at return to work, special electives could be added to the residency program in the form of didactic blocks, such as community outreach programming, administrative or practice management training, or focused research projects [15]. Although support for childbearing during training had little or no influence on the choice of program for most respondents in both periods, there was a trend in period 2 showing support was becoming more of a factor. This indicates that more prospective residents are considering these benefits when selecting a program. Thus, it may benefit programs to highlight issues around pregnancy during recruitment without fear of a negative effect on enrollment.

Nearly one-quarter of respondents were unaware of a formal parental leave policy, which is concerning and indicates an area for increased education. During period 1, the institution’s policy provided two days of paid parental leave for new fathers and six weeks of paid leave for new mothers, with an option of an additional six weeks unpaid. During period 2, paid parental leave increased to five days, with no changes to other forms of leave. The parental leave policy could be viewed as a high priority for change. Encouragingly, our institution implemented four weeks of parental leave for fathers less than a year after the completion of the period 2 survey.

Strengths of this study include a) use of the same survey instrument for this longitudinal comparative analysis and b) the breadth of Graduate Medical Education (GME) programs represented within this study in contrast to many other studies that have focused only on single specialties. Our study also included the attitudes/experiences of residents who were pregnant (female residents), male residents who became fathers during training, and residents not have pregnancies, thereby providing a more comprehensive view of the effects of pregnancy on the GME experience. The generalizability of this study may be limited by a relatively low-response rate and potential responder bias, i.e., residents who had particularly strong experiences regarding pregnancy during training may have been more likely to complete the survey. This responder bias was thought to be counterbalanced by the potential for non-pregnant trainees to share their dissatisfaction with covering for pregnant trainees due to lost elective time, additional shifts/calls, or limited to no payback for time worked. Residencies in other countries within Africa and Asia may also be very different and may not be comparable to our study population.

## Conclusion

Our study underscores the importance of proactively addressing issues surrounding pregnancy during residency. The majority of participants characterized pregnancy during their training to be somewhat or very difficult. Pregnancy complications were substantially higher among residents and for pregnant partners of residents compared to the general population. Our findings should serve as an impetus to discuss guidelines surrounding pregnancy during training, as well as focus on efforts to try to identify the factors associated with increased high pregnancy complication rate in residents and implement solutions that could improve attitudes and outcomes for individuals, programs, and institutions.

## Data Availability

Full data are available by request.
